# Lower Body Kinematics Monitoring in Running Using Fabric-Based Wearable Sensors and Deep Convolutional Neural Networks

**DOI:** 10.3390/s19235325

**Published:** 2019-12-03

**Authors:** Mohsen Gholami, Ahmad Rezaei, Tyler J. Cuthbert, Christopher Napier, Carlo Menon

**Affiliations:** Menrva Research Group, Schools of Mechatronic Systems Engineering & Engineering Science, Simon Fraser University, Metro Vancouver, BC V5A 1S6, Canada; mohsen_gholami@sfu.ca (M.G.); ahmad_rezaei@sfu.ca (A.R.); tyler_cuthbert@sfu.ca (T.J.C.); cnapier@sfu.ca (C.N.)

**Keywords:** strain sensors, running, convolutional neural networks, gait analysis, kinematics

## Abstract

Continuous kinematic monitoring of runners is crucial to inform runners of inappropriate running habits. Motion capture systems are the gold standard for gait analysis, but they are spatially limited to laboratories. Recently, wearable sensors have gained attention as an unobtrusive method to analyze performance metrics and the health conditions of runners. In this study, we developed a system capable of estimating joint angles in sagittal, frontal, and transverse planes during running. A prototype with fiber strain sensors was fabricated. The positions of the sensors on the pelvis were optimized using a genetic algorithm. A cohort of ten people completed 15 min of running at five different speeds for gait analysis by our prototype device. The joint angles were estimated by a deep convolutional neural network in inter- and intra-participant scenarios. In intra-participant tests, root mean square error (RMSE) and normalized root mean square error (NRMSE) of less than 2.2° and 5.3%, respectively, were obtained for hip, knee, and ankle joints in sagittal, frontal, and transverse planes. The RMSE and NRMSE in inter-participant tests were less than 6.4° and 10%, respectively, in the sagittal plane. The accuracy of this device and methodology could yield potential applications as a soft wearable device for gait monitoring of runners.

## 1. Introduction

Running kinematics are important biomechanical parameters that are associated with injury risk and running economy [1,2]. Previous studies have been conducted on runners on the relationship between different kinematic parameters [3]. The range of motion of the hip joint in the frontal plane during running has been correlated with a risk of injuries of the pelvis, hip, and knee [2]. Foot and shank angles at the initial ground contact, along with knee and hip range of motion during the stance phase are related to running performance [1]. The ankle joint angle at initial contact has also been related to several kinetic risk factors for running-related injuries [4]. Therefore, continuous multi-axis kinematic monitoring of lower extremities is an important consideration for the prevention of running injuries and performance improvement. Sophisticated gait analysis, conducted in clinics, provides the most accurate results, but this solution is not practical for long-term monitoring of runners during daily training. Gait laboratories are also not accessible to the majority of recreational runners. An alternative solution to this problem has been the development of wearable sensors to measure running kinematics.

Inertial measurement units (IMUs) are the most common wearable sensor systems. IMUs have been used to measure kinematics and kinetics of the lower body including two- and three-dimensional joint angles [5,6,7,8], changes in the kinematics [9], ground reaction forces [10], and lower body joint power [11]. IMU-based wearable sensors have limitations when measuring the multi-axis joint angle. Magnetometers are susceptible to ferromagnetic disturbances [6], and therefore heading drift in the IMUs is a challenge. The physiological bone coordinate system is also different from the IMU’s coordinate system attached to each segment which can lead to a measurement error [8]. Thus, the correlation between angles measured by IMUs and a motion capture system have been reported to be poor for the hip frontal plane [12]. Considering the accuracy of IMUs for joint angle measurement, it should be highlighted that a large number of the previous works have attached the motion capture markers to the IMU units for measuring reference joint angles [5], however, attaching markers to anatomical bone marks, which is the common method in gait analysis, can significantly increase the error [13]. 

Soft strain sensors are an alternative solution for human motion monitoring that have been employed for lower body monitoring [14,15,16,17], trunk angle measurement [18], gait phase detection [19], and posture classification [20]. The primary advantage of fabric-based strain sensors as compared with rigid IMUs is flexibility and comfort during their use. Strain sensors have been shown to measure hip, knee, and ankle joint angles in the sagittal plane with a root mean square error (RMSE) of 6° [16], 2° to 15° [15,16,17], and 3° to 10° [16,17], respectively. The peak knee flexion angle during gait was also estimated with an RMSE less than 2° [21]. Totaro et al. [17] extended the use of strain sensors for lower body monitoring from the sagittal plane to the frontal and transverse planes for only the ankle joint, however, the performance of the sensors for non-sagittal plane angle measurements during walking and running was not investigated. Mengüç et al. [16] introduced the use of stain sensors to monitor running in the sagittal plane. While an RMSE of 2° to 10° was reported for a lower body joint measurement during walking and simple joint flexion tasks [15,16,17], the error was shown to be less than 15° for running [16]. A comparison of the performance of strain sensors for running [16] with walking [16] and joint bending exercises [15,17] highlights the challenges of this technology in running applications. 

A primary problem of soft strain sensors is the calibration of sensors for each individual person. Methods such as linear fitting [16,17], Fourier series [15], machine learning (random forest and neural network) [14], and deep learning algorithms (LSTM) [22] have been previously reported addressing calibration issues. In a previous study, we showed that a machine learning model decreased the error of knee angle measurements approximately 3° in contrast to a linear regressor [14]. Incorporating a deep learning model has also been shown to lower the error of full-body motion tracking compared with linear models [22,23].

Although calibrating a sensor to a specific person leads to high accuracy, it requires a gold standard motion capture system. An alternative approach is to introduce a model that is calibrated on several people and can be used by others. In previous works, a random forest regressor obtained an RMSE of 7° for the knee angle measurements in an inter-participant test [14]. Upper body postures were also classified with an accuracy of 65% in an inter-participant scenario [20]. Changes in the position of sensors on the body and differences in body shapes are some challenges in inter-participant tests.

Considering the need for the use of wearable sensors (IMUs or flexible strain sensors) for running kinematic monitoring, reliability and opportunities of IMUs have been investigated for indoor and in-field applications [24,25]. Previous works related to soft strain sensors have focused on sensor development [26] and their application for tracking simple activities such as joint bending [26] and two-dimensional kinematics of the gait during running [16]. Herein, this work addresses the validation of a fiber-based strain sensor for three-dimensional kinematic monitoring during running. Improvements in measurement accuracy were addressed by optimization of sensor placement and advanced signal processing. 

## 2. Materials and Methods

### 2.1. Strain Sensor

The fiber sensors that were incorporated into the prototype were produced as previously reported [27]. A multifilament spandex yarn (polyether, urethane, and urea) was dip coated with a carbon black thermoplastic elastomer composite. A solution consisting of Hytrel 3078 (H3078, DuPont Kingston, ON) in dichloromethane (5 wt % H3078) and carbon black (50 wt % with respect to H3078) was used to coat the spandex yarn using a continuous roll-to-roll method at a rate of 3.81 cm/second and wound onto a bobbin 1.83 meters from the exit of the solution (to allow sufficient drying prior to collection to reduce fibers sticking together). Any remaining solvent was removed in vacuo at 60 °C for 30 min. Prior to use, all sensors were conditioned by straining to 40% 100x at 10% per second with a sinusoidal wave pattern. To protect the sensor from shorting when exposed to conductive liquids (such as sweat), an additional insulating sheath was applied by dip coating in a 5 wt % poly(styrene-b-ethylene-co-butadiene-b-styrene) cyclohexane solution once connections were made to the connecting wires. The analysis of the sensors range, gauge factor, hysteresis, long-term sinusoidal testing, strain rate, and long-term random testing were reported in a previous study [27]. The sensor had no hysteresis below 30% strain, consistent signal over 4 hours, and a gauge factor of 5. Strain response of the sensor at 10%, 20%, and 30% strain were linear and consistent. The piezoresistive sensing was limited to 90% strain, while the working range was limited to 30% to ensure signal linearity and limit plastic deformation. The performance of the sensor strained at different frequencies has been included in this study (Appendix A). The sensors were able to track up to 10 Hz (200% per second; 100 mm/s for a 50 mm sample).

### 2.2. Optimization of Sensor Placement

Optimization of sensor placement is an important step to determine the minimum number of sensor positions that lead to the highest accuracy. Optimization was only studied for the hip joint since it requires monitoring three degrees of freedom with many possible positions and orientations that could affect the sensor performance. The knee and ankle have limited degrees of freedom and potential positions. Therefore, sensors were placed on the ankle and knee empirically based on the primary axis of movement and refined with trial and error.

Sensor placement for the hip joint angle was completed with the goal of finding the combination of positions that led to the highest accuracy in the joint angle estimation. The positioning was treated as a feature selection problem, and positions were considered as features according to the method introduced in a previous work [28]. The strain at all possible positions was measured using optical cameras and reflective markers. The objective function was a linear regressor’s accuracy (R^2^ error) that estimated joint angles in the sagittal, frontal, and transverse planes in a leave-one-person-out cross-validation. Two methods were employed to solve the feature selection problem: a forward sequential (FS) [29] method and a genetic algorithm (GA) [30]. There are two main categories of feature selection methods: filter and wrapper. Filter methods use statistical tests to find the best feature subset without training a machine learning model. Wrapper methods evaluate the usefulness of a feature subset by training a machine learning model and computing the corresponding accuracies [31]. The wrapper method has been reported to find better feature subsets as compared with filter methods because it considers the accuracy of any feature subset for the prediction problem. Therefore, wrapper methods were employed in this study. Two main search strategies used for wrapper methods are sequential algorithms and randomized algorithms [31]. In this study, a forward sequential algorithm from the sequential algorithms and a genetic algorithm from randomized algorithms were implemented. A linear regression model was used as the machine learning algorithm which previously has achieved satisfactory accuracies in joint angle estimation using strain data while being less computationally demanding as compared with more complex machine learning algorithms [14]. 

#### 2.2.1. Data for Sensor Placement

**Figure 1:**
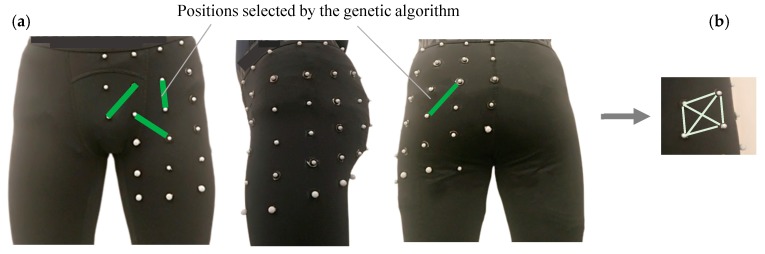
(**a**) Grid of markers on garment for optimization of sensor position on pelvis. Initial vertical and horizontal distances of markers were 4 cm before wearing the tight and (**b**) all potential sensor orientation including horizontal, vertical, and two diagonals were considered.

To find the best positions and number of sensors to monitor the hip joint angle, strains on the garment during running were recorded using six motion capture cameras (Vicon, Oxford, UK). A total of 50 motion capture markers (diameter of 6 mm) were placed on tights 4 cm apart in a grid pattern (Figure 1). Five participants (all male, age 25 ± 2 years, weight 75 ± 7 kg, and height 180 ± 2 cm) participated in the data collection. The experimental protocol was approved by the Office of Research Ethics at Simon Fraser University. Prior to any data collection, written informed consent was obtained from all participants. Data recording was completed at the following three speeds: 8 km/h, 10 km/h, and 12 km/h, each for two minutes. The running test was conducted on a split-belt instrumented treadmill (Bertec Corporation, Columbus, OH, USA).

The distances of neighboring markers in vertical, horizontal, and both diagonal directions were computed using the three-dimensional marker positions (Figure 1b). Strains on the garment were computed using the following formula:

(1) $$strain=\frac{L-L_{0}}{L_{0}},$$

where *L* was the distance of markers and $L_{0}$ was the initial distance of markers in a neutral standing posture $(L_{0}$ was greater than or equal to 4 cm). When the garment was worn, the fabric had a natural stretch, and therefore was able to increase or decrease in length. If the garment stretch decreased such that wrinkles were formed, and effectively resulting in data that was not ”real”, a limit was created so that this data would be excluded. Therefore, during the kinematic tracking, the value of *L* was limited to a value greater than or equal to 4 cm, which was determined to be a greater length than when wrinkles would form. A condition was applied such that each sensor would strain a minimum of 10% (relative to its starting position) during the intended running motion to maximize the obtained signal and accuracy. This excluded any sensor positions that would not strain during the running motion. Twelve reflective markers were used to define the pelvis and thigh segments to measure the hip joint angle during the test. The marker set and segments are discussed in the next sections. 

#### 2.2.2. Sequential Forward Method

In this method, we started from an empty subset of positions (features) and added the next position to the already selected positions, which led to the highest value of the objective function [29]. The method is described in Algorithm 1 (Appendix B) and was implemented in Python. In the pseudocode, Y is the feature set that contains all features, $X_{k}$ is the feature subset selected by the algorithm after k iterations and contains k features, J is the objective function that we aim to maximize, and $x^{+}$ is a new feature that is selected to be added to the current feature subset ($X_{k}$) in each iteration. 

#### 2.2.3. Genetic Algorithm Method

The genetic algorithm has been frequently used as a feature selection for machine learning problems [32]. A custom genetic algorithm model was implemented in Python for this study [30]. The pseudocode of the genetic-based feature selection algorithm is described in Algorithm 2 (Appendix B). 

There are two different approaches for the implementation of a genetic algorithm: decimal and binary. In the binary GA, each candidate will be represented in a binary format with zeros and ones. Each candidate in the search domain will be called a chromosome and each zero or one in a chromosome is called a gene. The genetic algorithm has three major steps. First, a population of candidates is generated. Second, a mating pool from the population is selected. Third, each time two candidates from the population pool are selected, “cross-over” and “mutation” operations are completed [30]. In a “cross-over” operation two chromosomes that are selected exchange some genes with each other and in “mutation” one gene of chromosomes changes from zero to one or from one to zero. Both cross-over and mutation have different methods of implementation and are completed by a probability defined in the algorithm. This procedure continues and, at each iteration, a new generation is produced with the same size as the initial population. It is expected that generation evolves in this procedure and better individuals are produced based on the biological concept of GA [30].

In this study, an initial population of potential sensor positions was made randomly containing 30 individuals. Individuals of the population were binary chromosomes with a length of 40, equal to the number of all possible sensor positions. In the binary chromosome, a gene with a value of one indicated the presence of a sensor position with the same index and a gene with a value of zero indicated the absence of the corresponding position. A mating pool containing 15 individuals was then selected using a roulette wheel selection method. A couple from the mating pool were selected randomly and one-point crossover applied by the probability of Pc = 0.8. The crossover point was selected randomly and under the condition that exchanging parts had the same number of zeros and ones. Consequently, children had the same number of zeros and ones as their parents and the algorithm was guaranteed to find the best feature subset with a desired number of features. The generated offspring were mutated by the probability of Pm = 0.5. The crossover and mutation probability were tuned to achieve the best accuracy. Using the interchange mutation method, a random gene of one and a random gene of zero were selected and their values were swapped. In order to guarantee that the best individuals were continued in the next generations, elitism was applied. The best two individuals of a generation were moved to the next generation. 

#### 2.2.4. Sensor Placement Results

**Figure 2:**
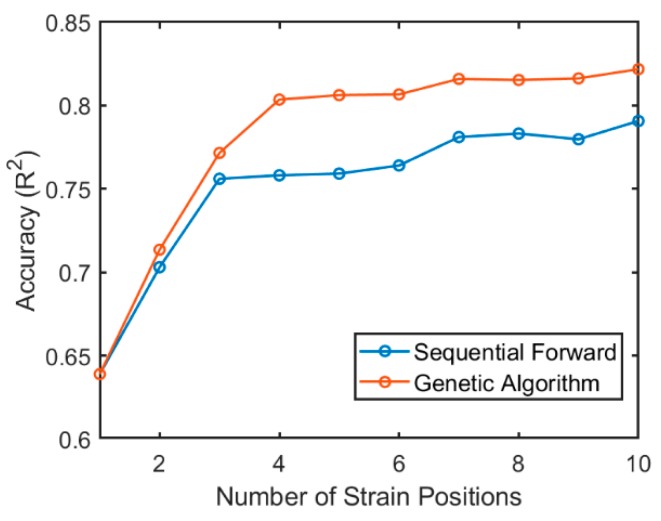
Inter-participant accuracy of estimating 3D hip angle using the best position subset at different sizes.

The best feature subset at different sizes was found using both sequential forward and genetic algorithm methods. Average R^2^ of inter-participant estimated angles using the best feature subset with different sizes are shown in Figure 2. The genetic algorithm could find a better feature subset as compared with the sequential forward method. The accuracy of the feature subset found by GA did not significantly improve after the position subset size of four, and therefore this enabled us to use the least number of sensors to reduce power consumption and decrease the device production time. Selected positions are shown in Figure 1, indicated with a green line.

### 2.3. Prototype

**Figure 3:**
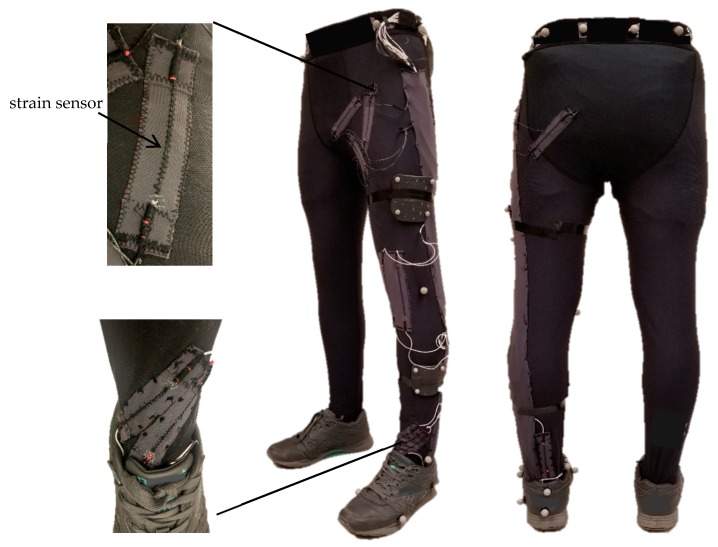
The fabric-based strain sensors and motion tracker markers mounted on the left leg.

Both ends of the strain sensors were connected to wires by conductive materials. Sensors were then fixed on the garment by stitching over the sensor. Sensors on the pelvis area were placed in the specified positions determined by the genetic algorithm. Sensors on the knee and ankle were placed empirically, as shown in Figure 3. All sensors were mounted on the left leg of a tight legging as follows: four sensors on the pelvis, two sensors on the knee (on the front side), and three sensors on the ankle (two on the front side and one on the back side). For simplification of data analysis with our proof-of-concept device, we chose to only study one leg. Monitoring both legs is feasible with a symmetrical system (on both legs) which would be implemented for a commercial product and would allow the study of leg dependence on gait and running efficiency, although this will not be discussed here. 

### 2.4. Experimental Setup

All sensors mounted on the prototype were connected to a data acquisition system (DAQ) to read changes in the sensors’ resistance. The sensors’ resistance was read by a voltage divider circuit. The resistor used in the circuit was 40 kΩ. 

Six motion capture cameras (Vicon, Oxford, UK) were used to capture 3D kinematic data from 23 reflective markers mounted on the lower body of participants who were running on a split-belt instrumented treadmill (Bertec Corporation, Columbus, OH, USA). Motion capture data was synchronized with sensor signals using a synchronization signal. Motion capture data was recorded using Vicon Nexus software. The data from strain sensors and motion capture system were recorded at a sampling rate of 100 Hz. 

### 2.5. Participant and Data Collection Protocol

Ten healthy male participants (age 27 ± 4 years, height 177 ± 7 cm, and weight 72 ± 7 kg) were recruited for this study. The experimental protocol was approved by the Office of Research Ethics at Simon Fraser University. Prior to any data collection, written informed consent was obtained from all participants.

The protocol for data collection was a total of 15 min of running consisting of 15 one-minute trials at the following five speeds: 8, 9, 10, 11, and 12 km/h. Participants started from the slowest speed and progressed to the fastest speed. This was repeated three times. Speeds were not randomized since it was more convenient for participants to start from the slowest speed and increase sequentially to the fastest speed. 

### 2.6. Measuring Reference Lower Body Kinematics

Twenty-three reflective motion capture markers were mounted on the lower body to monitor lower body kinematics. The pelvis segment was defined following the CODA segment by four static markers, right and left anterior superior iliac spine and right and left posterior superior iliac spine (R/L PSIS). Four markers were also used as tracking markers of the pelvis including R/L PSIS and two additional markers on the iliac crest. The thigh segment was defined using a proximal marker on the greater trochanter and the hip joint center (defined by the pelvis segment) and two distal markers on the knee (lateral and medial epicondyle of the femur). Four reflective markers fixed on a plate were mounted to the thigh segment as the tracking markers. The shank segment was defined using two proximal markers (lateral and medial epicondyle of the femur) and two distal markers (lateral and medial ankle malleoli). A plate with four markers on it was mounted on the shank segment as the tracking markers. The foot segment was defined by two proximal markers (lateral and medial ankle malleoli) and two distal markers (head of 1^st^ and 5^th^ metatarsals). After capturing the static trial all static markers were removed and only tracking markers were used for the running test.

Marker trajectory data were recorded by Vicon Nexus software. Motion capture data were imported to Visual 3D software (C-Motion, Inc., Germantown, USA) and a musculoskeletal lower body model was created. A virtual segment method was used to calibrate actual joint angles according to the recommendation of Visual 3D [33]. Joint angles were filtered using a 4th order Butterworth filter with a cutoff frequency of 6 Hz, following previous recommendation [34]. 

### 2.7. Data Preprocessing

The signal of the strain sensors during the static standing posture at the beginning of the trials was used to compute

(2) $$\frac{\Delta V}{V_{0}}=\frac{V-V_{0}}{V_{0}},$$

where V is the voltage of strain sensors during the test and $V_{0}$ is the initial voltage of strain sensors before the test in the static standing posture. In the following, $\Delta V/V_{0}$ was used to estimate actual angles. Data obtained from the strain sensors were then normalized by subtracting the mean values and dividing by standard deviation. Training and testing datasets were normalized separately to keep the test data unseen. Data were then smoothed using a Savitzky-Golay filter [35] with a window length of 31 frames and an order of 5. Parameters of the filter were selected empirically to eliminate noise and keep the primary patterns of signals.

### 2.8. Convolutional Neural Network Architecture 

**Figure 4:**
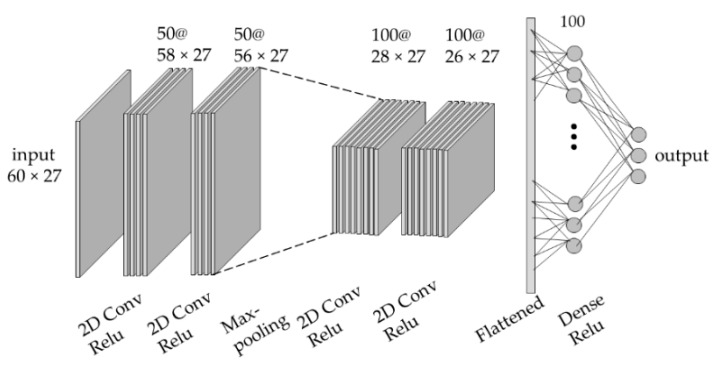
Architecture of the CNN model for inter-participant tests. A similar CNN model was used for intra-participant tests with six outputs.

A four-layer convolutional neural network (CNN) was implemented in Keras backend with TensorFlow [36]. The architecture of the two-dimensional CNN model is depicted in Figure 4. The input of the CNN model was the raw signal extracted from 600 ms (60 data points at 100 Hz) time-window moving on all 9 sensors of the prototype and the first and second derivatives of the raw signal. 

The input shape of the model was 60 × 27. The output of the CNN model for the intra-participant tests were the following six joint angles: hip sagittal plane, hip frontal plane, hip transverse plane, knee sagittal plane, ankle sagittal plane, and ankle frontal plane angles. The output of the model for the inter-participant tests was the following 3 angles in the sagittal plane: hip, knee, and ankle. 

The linear function ReLU activated all convolutional and dense layers and Xavier uniform function initialized layers. The kernel shape was (3,1), which meant that kernels moved on the time dimension of layers. The batch size was 128 and training stopped after 10 epochs as the model started overfitting on the training data. Training the model on all training data for inter-participant tests led to a bigger generalization error as compared with using a subsample of data. Therefore, 60 percent of the training data were randomly sampled with replacement (one data point could be selected multiple times) and used to train the CNN model. Subsampling with replacement is a technique used in the bagged models [37]. 

In intra-participant models the loss function was the MSE of the predicted 6 outputs, which was calculated by the following formula:

(3) $$MSE\;=\;\frac{1}{6N}\sum_{i=1}^{N}\sum_{j=1}^{6}{\left({\hat{y}}_{i,j}\;-\;y_{i,j}\right)}^{2},$$

where ${\hat{y}}_{i,j}$ is the predicted angle, $y_{i,j}$ is the actual angle, N is the number of samples, and 6 is the number of outputs. In inter-participant models, since the estimation error of ankle and hip were bigger than the knee, the loss function was defined by weight parameters for ankle and hip as follows:

(4) $$\begin{array}{c} MSE\;=\;A\times \frac{1}{N}\overset{N}{\underset{i=1}{\sum }}{\left({\hat{y}}_{i,Hip}-y_{i,Hip}\right)}^{2}\\ +B\times \frac{1}{N}\overset{N}{\underset{i=1}{\sum }}{\left({\hat{y}}_{i,Knee}-y_{i,Knee}\right)}^{2}+C\times \frac{1}{N}\overset{N}{\underset{i=1}{\sum }}{\left({\hat{y}}_{i,Ankle}-y_{i,Ankle}\right)}^{2}, \end{array}$$

where *A*, *B*, and *C* were weights defined to adjust the parameters to improve the accuracy of the worst estimated joint angles. *A*, *B*, and *C* were empirically selected to be 2, 1, and 5, respectively. 

### 2.9. Evaluation Method and Metrics

The CNN model was trained to estimate the lower body kinematics computed by Visual 3D in both inter- and intra-participant scenarios. In the intra-participant scenario, a CNN model was trained and tested using data of each specific individual, while in the inter-participant scenario a CNN model was trained based on data of several participants and was tested for a separate participant. The advantage of the inter-participant scenario is the elimination of calibration for new people. 

From each participant, 15 min of running data at 100 Hz was collected. Consequently, 90,000 data samples from each of the 9 sensors across 10 participants were used as the dataset. Since the dataset contained three trials from each speed, in the intra-participant scenario, the dataset was split into three folds, each containing 5 min of running at five different speeds. Then 3-fold cross-validation was applied to the data; each time, two folds were used as the training and the remaining fold was considered as the test data. This procedure was repeated until all folds were used as the test data. The average accuracy over three folds was reported. In inter-participant tests, data of one participant was used as the test data and data of the remaining 9 participants were used for training. This procedure was repeated until all participants were used as the test data. The accuracy of the model in both inter- and intra-participant scenarios was averaged among all participants. In order to evaluate the performance of the inter- and intra-participant models at different speeds, the same training data, which has been explained above (included all speeds), were used but the test set was merely a specific speed of running. The coefficient of determination (R^2^), root mean square error (RMSE), and normalized root mean square error (NRMSE) of estimated values were used to evaluate the performance of the models. 

## 3. Results

### 3.1. Intra-Participant Results

**Figure 5:**
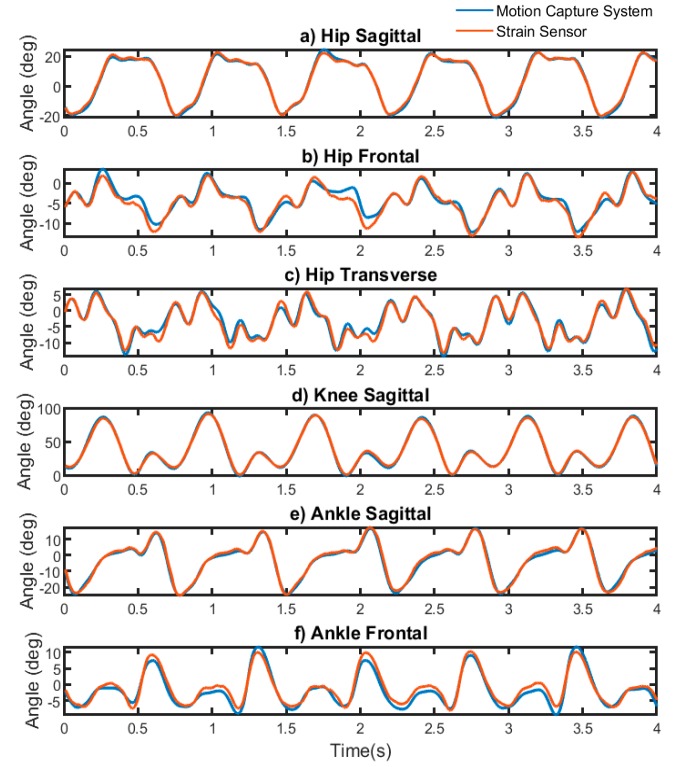
An example of angles measured by strain sensors in intra-participant tests versus angles measured by motion capture system in (**a**) hip sagittal plane, (**b**) hip frontal plane, (**c**) hip transverse plane, (**d**) knee sagittal plane, (**e**) ankle sagittal plane, and (**f**) ankle frontal plane.

The average accuracy of the CNN model for intra-participant tests is shown in Table 1. The RMSE and NRMSE of the estimated lower body joint angles at different planes were less than 2.2° and 5.25%, respectively. The estimated angles as compared with angles measured by motion capture systems are shown in Figure 5. Joint angles in frontal and transverse planes were more complex as compared with the sagittal plane. The range of motion in the sagittal plane was bigger than the non-sagittal planes (Figure 5).

**Figure 6:**
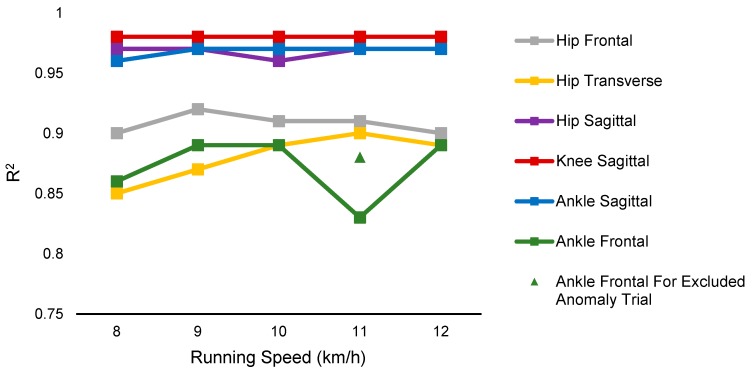
Comparison of the R^2^ error of the estimated angle at different speeds in the intra-participant evaluation. The performance of the sensor and algorithm were consistent at different speeds.

The average accuracy for the different speeds is shown in Figure 6 and Table A1 (Appendix C). There was an anomaly in the ankle frontal data of one participant at 11 km/h. The dataset recorded from each participant consisted of 15 trials, i.e., three trials at each of the five speeds. One trial of one of the participant’s ankle frontal data at 11 km/h did not match the data at all other trials. An additional point has been added in Figure 6, which excludes this trial, and indicates a consistent performance at different speeds of ankle frontal plane.

### 3.2. Inter-Participant Results

**Figure 7:**
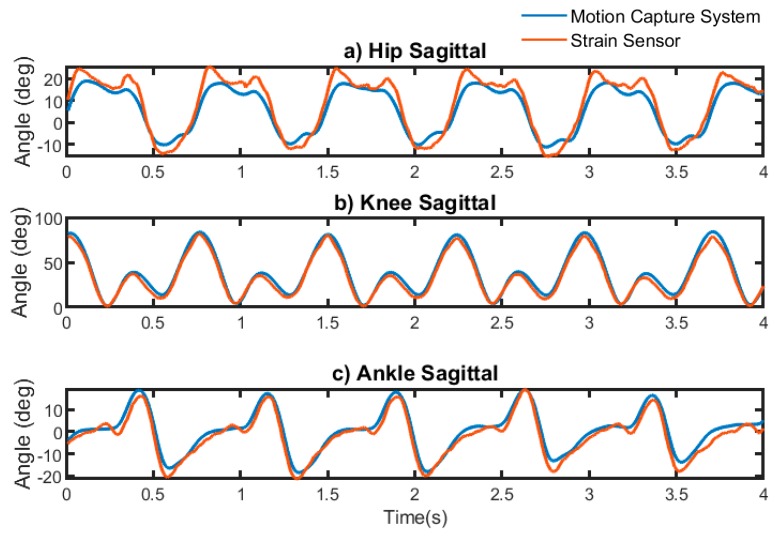
An example of angles measured by strain sensors in inter-participant tests versus angles measured by motion capture system in (**a**) hip sagittal plane, (**b**) knee sagittal plane, and (**c**) ankle sagittal plane. Pattern and range of motion of the ankle and hip were less consistent among participants as compared with pattern and range of motion of the knee.

**Figure 8:**
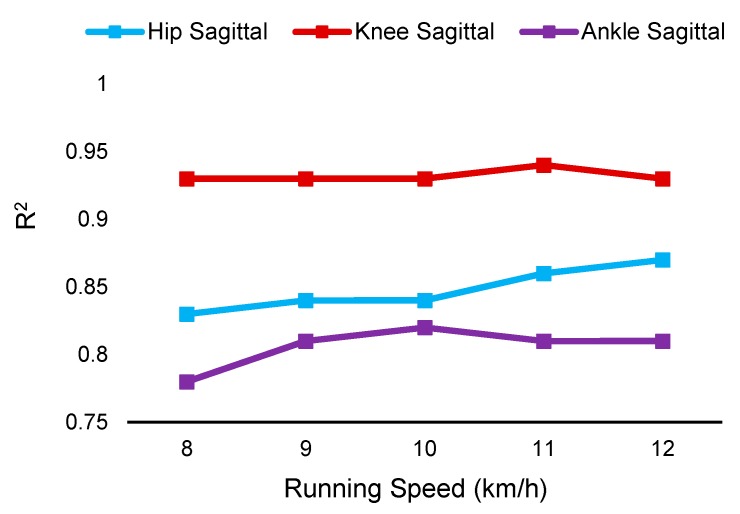
Comparison of the R^2^ error of the estimated angle at different speeds in inter-participant evaluation. The performance of the sensor and algorithm were consistent at different speeds.

In this method, one person was considered as a test and the remaining persons were used as the training dataset. The average accuracy of the models is reported in Table 2. The estimated angles as compared with angles measured by motion capture systems are shown in Figure 7. The RMSE and NRMSE of estimated lower body joint angles at different planes were less than 6.5° and 10% for inter-participant models. An example of estimated angles as compared with the angles measured by the motion capture system is shown in Figure 6. The accuracy of the inter-participant models for different speeds is also shown in Figure 8 and Table A2 (Appendix C). In this section models are the same models trained for inter-participant tests, but the test data were split into different speeds. Then, the accuracy and error of the model at different speeds of test data were measured.

## 4. Discussion and Conclusions

In this work, we developed a fabric-based wearable sensor and used a deep convolutional neural network to estimate lower body kinematics in sagittal, frontal, and transverse planes. A fiber-based strain sensor coated with insulating sheath was used for prototyping [27]. The positions of sensors on the pelvis were optimized using two methods, a genetic algorithm and a sequential forward method. Optimization of the sensor positioning based on the strain pattern of the garment was introduced in a previous study for upper-body monitoring [28]. In this study, accuracy was the objective function, and the number of sensors was defined as the input to the algorithm. Therefore, a better comparison of the best accuracies using different numbers of the sensor was made (Figure 2). Optimization was employed to maximize the accuracy (R^2^) in inter-participants scenarios since training the inter-participant CNN model was more challenging. A comparison of the results of the sequential forward method and genetic algorithm showed that the later search method found better feature subsets. GA had some limitations including that the results were dependent on the initial population and on the parameters of the algorithm. The parameters of the genetic algorithm such as population size, selection method, cross-over and mutation probability, elitism, and fitness function were selected and tuned to make the algorithm more robust. The comparison of the performance of the GA with a deterministic search method (FS) showed that the GA parameters were selected effectively. The sequential forward method has some limitations including the inability to delete features selected in the previous iteration, at the next iteration; this inability hinders the search domain of the algorithm resulting in many combinations of positions not being evaluated. 

A comparison with previous studies shows that we have expanded the lower body monitoring from only sagittal plane to include frontal and transverse planes during running. In the intra-participant evaluation, estimated values in the sagittal plane were more accurate as compared with other planes. The lowest R^2^ of 0.97 was obtained for the sagittal plane while the lowest R^2^ of 0.88 was obtained for the frontal and transverse planes for person-specific models. Figure 5 demonstrates that there is a greater difference between angles measured by strain sensors and motion capture in hip frontal, hip transverse, and ankle frontal planes. We reasoned that the difference of accuracy in sagittal and non-sagittal planes was from the complexity of movements, smaller angles that require detection, and the sensor and CNN’s ability to separate these smaller angle changes in the frontal and transverse planes from the larger sagittal plane. Since the range of motion in the sagittal plane was larger than other planes, sensors were affected more with flexion-extension than abduction-adduction and rotation during running. Therefore, the sensor’s signal was highly correlated with sagittal angles and less correlated with non-sagittal angles. 

**Figure A1:**
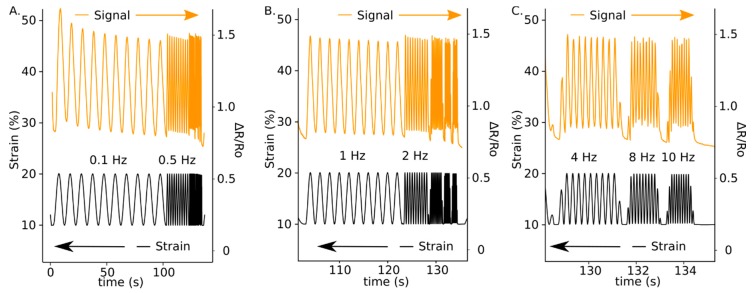
(**A**–**C**) Sinusoidal strain wave pattern (270° phase change, from 10% to 20% strain) at varying frequencies (0.1, 0.5, 1, 2, 4, 8, and 10 Hz) overlaid with the resulting resistance from a typical sensor used in the wearable device.

A previous study showed that errors in estimation increased at higher speeds [16]. In contrast, in this study, similar accuracy at fast and slow speeds was achieved (Figure 6 and Figure 8) thanks to the deep convolutional neural network and sensor characteristics [27]. According to the sensor characteristics of frequency and strain rate, the sensor was able to track at low and high frequencies (up to 10 Hz, Figure A1 and Appendix A) [27]. The CNN models can better predict nonlinear patterns (hysteresis and time-dependent behavior that are inevitable in piezoresistive sensors at higher frequencies and strain rates) as compared with linear models used in the previous works, and therefore lower errors at higher speeds were obtained in contrast to previous work [16]. Figure 6 demonstrates a lower accuracy of ankle frontal tracking at the speed of 11 km/h as compared to the other speeds, even at the higher speed of 12 km/h. The decrease in accuracy for this trial was due to an anomaly in the dataset of one participant. The ankle frontal values changed such that it had a detrimental effect on the CNN model’s ability to predict this trial when it was assigned as the test set. It is worthy to note that this was directly correlated to a lower range of motion during the 11km/h speed and was not consistent with the participant’s ankle frontal range of motion in all other speed trials. This negatively affected the R^2^ value and, for comparison, we have included an extra data point that indicates the average for ankle frontal with this dataset removed, which is consistent with the other R^2^ values for frontal and transverse values (Figure 6). 

Validation of the gold standard optical motion capture system in measuring actual angles in frontal and transverse planes between participants has been elusive thus far [38]. Therefore, in inter-participant evaluation, the model was trained to estimate only sagittal plane angles. The patterns and range of motion of knee joint angles were more consistent among participants as compared with the pattern and range of motion of the ankle and hip joint angles, and therefore smaller errors were obtained in estimating knee angles. Figure 7 demonstrates that there is a greater difference between estimated and reference angles of the hip and ankle versus knee joint. The R^2^ and NRMSE of the estimated knee angles were 0.93 and 6.30%, respectively, while the ankle and hip angles were estimated with R^2^ and NRMSE of 0.81 and 9.9%, and 0.85 and 9.34%, respectively. The CNN model’s accuracy was lower when the pattern of joint angles in the test dataset was different from the patterns of joint angles in the training dataset. It can be expected that having a large dataset that includes different gait patterns would lead to a smaller error in the ankle and hip joint angle estimations. 

The results of this study had smaller errors as compared with previous works. Mengüç et al. used hyper-elastic sensors for sagittal plane monitoring of runners [16] and could obtain RMSE of less than 15°, 10°, and 6° for knee, hip, and ankle, respectively; while we have achieved RMSE of 1.12°, 2.2°, and 1.3° for knee, hip, and ankle joint, respectively. Capacitive strain sensors were used to measure the multi-axis ankle angle and have obtained an RMSE of less than 4° [17]; while in this study ankle joint in sagittal and frontal planes were measured with an RMSE of less than 1.56°. The method used in this work, including sensor placement and the convolutional neural network employed for angle estimation, outperformed previous studies. 

In this study, a CNN model was used for signal processing purposes. The advantage of CNN is the ability to automatically extract features from the input signal by convolutional layers. However, CNN models work better when a large-scale dataset is available. There are opportunities for using the fabric-based wearable device proposed in this study and CNN models for other purposes such as gait phase detection, rehabilitation monitoring, and Parkinson’s disease monitoring according to previous works [8,39]. The challenges of using wearable fabric-based sensors, as compared with other technologies such as IMU-based systems and camera-based systems, are variations in performance that are due to differences in body-shapes and sensor positions, garment drifts during usage, and designing the system to be washable. 

The limitations of this research include the diversity of the cohort, testing environment, and test lengths. Expansion of this to people of different sex, sizes, and ages would allow testing on the abilities of the CNN model to track accurately with limited vs. expansive training datasets. The testing was conducted in a laboratory setting using a treadmill to control the speed with short data recording times. Outdoor non-treadmill running could result in different gait patterns with different terrain. Testing periods were limited, and long-term testing would be useful to analyze the performance and fit of the garment over time. To enable this type of testing alternative data recording or wireless capabilities would be required.

This work has addressed the challenge of creating reliable and accurate soft-sensor wearable devices that can track kinematic motion comparable to motion capture devices. We have outlined the sensor fabrication focused on the intended working range and ability to withstand sweat, device design and production, and implementation of CNN to produce a complete device. Finally, we were successful in producing a reliable kinematic motion capturing device that uses deep learning architecture to generalize inter-participant data beyond what is currently available. 

## Figures and Tables

**Table 1:** The average accuracy of inter-participant CNN models among 10 participants.

	Hip	Hip	Hip	Knee	Ankle	Ankle
	Sagittal	Frontal	Transverse	Sagittal	Sagittal	Frontal
**R^2^**	0.97 (0.02)	0.91 (0.04)	0.89 (0.04)	0.99 (0.00)	0.97 (0.01)	0.88 (0.10)
**RMSE (deg)**	2.20 (1.09)	1.12 (0.17)	1.33 (0.17)	1.12 (0.17)	1.33 (0.16)	1.56 (0.49)
**NRMSE (%)**	4.57 (1.00)	5.02 (0.74)	4.91 (0.41)	1.06 (0.17)	3.05 (0.69)	5.25 (1.36)

**Table 2:** The average accuracy and error of inter-participant CNN models among 10 participants.

	Hip	Knee	Ankle
	Sagittal	Sagittal	Sagittal
**R^2^**	0.85 (0.11)	0.93 (0.04)	0.81 (0.11)
**RMSE (deg)**	5.39 (2.29)	6.38 (2.32)	3.92 (0.73)
**NRMSE (%)**	9.34 (3.41)	6.30 (1.90)	9.99 (2.92)

## Appendix B

**Algorithm 1** Sequential Forward Method	
	**Input:** $Y=\left\{y_{1},y_{2},\ldots ,y_{d}\right\}$
	**Output:**$X_{k}=\left\{x_{j}|\;j=1,2,\ldots ,k;x_{j}\in Y\right\}\;$, k< d
	Initialization from an empty subset: $X_{0}=\emptyset ,\;k=0$
1:	$x^{+}=\arg\max\left[J\left(X_{k}+x\right)\right],x\in Y-X_{k}$
2:	$X_{k+1}=X_{k}+x^{+}$
3:	K = k + 1
4:	Go to step 1

**Algorithm 2** Genetic-Based Feature Selection Method	
	**Input:***F* (number of features), *G* (number of generations), *P* (number of participants)
1:	**for***f* = 1 to *F*
2:	Make a random population that all individuals have *f* ones
3:	**for***g =1* to *G*
4:	**for***p =* 1 to *P*
5:	Train the regressor on data of all participants except participant *p*
6:	Calculate R^2^ error of the regressor on the participant *p*
7:	**end for**
8:	Fitness= average of R^2^ errors
9:	Generating Mating pool
10:	Cross over
11:	Mutation
12:	Elitism
13:	Produce the new generation
14:	**end for**
15:	output best chromosome
16:	**end for**

## Appendix C

The performance of the intra- and inter-participant CNN models at different speeds is described in Table A1 and Table A2, respectively.

**Table A1:** Accuracy of intra-participants models at different speeds.

		Hip Sagittal	Hip Frontal	Hip Transverse	Knee Sagittal	Ankle Sagittal	Ankle Frontal
**8 km/h**	**R^2^**	0.97	0.90	0.85	0.98	0.96	0.86
	**RMSE (deg)**	2.19	1.14	1.42	3.24	1.73	1.58
	**NRMSE (%)**	5.84	8.71	10.08	4.36	5.58	9.10
**9 km/h**	**R^2^**	0.97	0.92	0.87	0.98	0.97	0.89
	**RMSE (deg)**	2.08	1.04	1.34	3.14	1.51	1.48
	**NRMSE (%)**	5.07	7.61	9.21	4.05	4.67	8.39
**10 km/h**	R^2^	0.96	0.91	0.89	0.98	0.97	0.89
	**RMSE (deg)**	2.79	1.13	1.32	3.22	1.53	1.50
	**NRMSE (%)**	5.75	8.07	8.66	3.98	4.65	8.19
**11 km/h**	**R^2^**	0.97	0.91	0.90	0.98	0.97	0.83
	**RMSE (deg)**	2.29	1.14	1.30	3.35	1.70	1.64
	**NRMSE (%)**	4.86	8.10	8.10	3.90	5.01	8.93
**12 km/h**	**R^2^**	0.97	0.90	0.89	0.98	0.97	0.89
	**RMSE (deg)**	2.75	1.27	1.44	3.47	1.72	1.54
	**NRMSE (%)**	5.28	8.34	8.30	3.81	5.04	8.29

**Table A2:** Accuracy of inter-participants models at different speeds.

		Hip	Knee	Ankle
		Sagittal	Sagittal	Sagittal
**8 km/h**	**R^2^**	0.83	0.93	0.78
	**RMSE (deg)**	4.71	6.00	3.84
	**NRMSE (%)**	11.71	7.37	11.52
**9 km/h**	**R^2^**	0.84	0.93	0.81
	**RMSE (deg)**	4.93	6.20	3.76
	**NRMSE (%)**	11.25	7.33	10.29
**10 km/h**	**R^2^**	0.84	0.93	0.82
	**RMSE (deg)**	5.67	6.28	3.79
	**NRMSE (%)**	11.23	6.89	10.70
**11 km/h**	**R^2^**	0.86	0.94	0.81
	**RMSE (deg)**	5.56	6.38	4.00
	**NRMSE (%)**	10.27	6.82	11.12
**12 km/h**	**R^2^**	0.87	0.93	0.81
	**RMSE (deg)**	5.84	7.05	4.22
	**NRMSE (%)**	10.23	7.17	11.32
